# Research Progress of A20 in Acute Leukemia

**DOI:** 10.31661/gmj.vi.3869

**Published:** 2025-06-21

**Authors:** Hongxia Wu, Jun Bai, Qiong Fa, Ke Yang, YanHong Li

**Affiliations:** ^1^ Department of Nuclear Medicine, The Second Hospital of Lanzhou University, Lanzhou, China; ^2^ Gansu Key Laboratory of Hematology, Lanzhou University Second Hospital, Lanzhou, China; ^3^ Department of Nuclear Medicine, The 940th Hospital of Joint Logistics Support Force of Chinese People’s Liberation, China; ^4^ Department of Hematology and Oncology, Gansu Provincial Central Hospital, Lanzhou, China

**Keywords:** A20, Acute Leukemia

## Abstract

Acute leukemia (AL) is a malignant tumor originating from hematopoietic stem cells. Its outstanding feature is the abnormal proliferation and aggregation of clonal leukemia cells in bone marrow and other hematopoietic tissues. Although chemotherapy, targeted immunotherapy and hematopoietic stem cell transplantation have been widely used in clinic, there are still relapse and refractory cases in AL patients. Finding new therapeutic targets and screening prognostic molecules are of great significance for the treatment and prognosis of AL. A20 protein, also known as tumor necrosis factor α-induced protein 3 (TNFAIP3), is a key protein that negatively inhibits the activation of nuclear transcription factor kB (NF-κB) and plays an important role in anti-tumor immune and inflammatory response. In leukemia and lymphoma, A20 is often inactivated, mutated or deleted. Lack of A20 can significantly inhibit the surveillance function of immune cells and mediate tumor immune escape. Therefore, exploring the mechanism of A20 in AL may have important research value and clinical significance for the treatment of AL. The purpose of this paper is to review the research progress of A20 in acute leukemia, and provide new theoretical basis and reference value for the pathogenesis research and targeted therapy of leukemia.

## Introduction

Acute leukemia (AL) is a biologically heterogeneous group of hematologic malignancies originating from hematopoietic stem or progenitor cells, characterized by the uncontrolled proliferation and accumulation of immature white blood cells in the bone marrow and peripheral blood. Based on the lineage of origin, AL is classified into acute lymphoblastic leukemia (ALL), which includes B-cell (B-ALL) and T-cell (T-ALL) subtypes, and acute myeloid leukemia (AML). ALL is more commonly observed in children, whereas AML predominates in the elderly population [1][2][3]. In recent decades, advancements in chemotherapeutic strategies, along with enhanced supportive and symptomatic care, have significantly improved the initial response rates to induction therapy. Currently, approximately 80-90% of patients with ALL and AML can achieve complete remission after the first cycle of induction chemotherapy [4][5]. However, relapse remains a significant clinical challenge, occurring in 30-40% of patients, even among initial responders [6]. Given this relapse risk and the complexity of disease heterogeneity, early and accurate assessment of prognosis and dynamic monitoring of disease progression are crucial for tailoring individualized treatment regimens. Furthermore, regular evaluation of immune function during treatment can help identify patients at higher risk of complications, enabling timely interventions [7]. In addition to optimizing current treatment protocols, there is a pressing need to identify novel biomarkers and therapeutic targets to better stratify patients, predict outcomes, and enhance treatment precision. This is particularly important in leukemias, where minimal residual disease (MRD) and circulating tumor DNA (ctDNA) detection via liquid biopsy techniques have shown promising implications for prognosis and relapse monitoring [8][9].

## 1. Pathogenic Factors of Acute Leukemia

### 1.1 Congenital Factors

Recent studies have highlighted that some types of leukemia may originate during intrauterine life. Observations in monozygotic twins developing identical subtypes of leukemia support the theory of a prenatal origin. In pediatric acute lymphoblastic leukemia (ALL), chromosomal abnormalities are identified in 60-70% of cases, with notable translocations such as t (4;11) (q21; q23), which is often associated with mixed lineage leukemia. Molecular analyses have confirmed the presence of fusion transcripts like MLL-AF4 in neonatal blood samples of individuals who later developed ALL, suggesting a prenatal initiation. Furthermore, genetic screening has identified other fusion genes such as TEL-AML1 and AML1-ETO in umbilical cord blood, both of which are associated with a significantly increased risk of leukemia [10][11].

### 1.2 Fusion Genes and Hereditary Predisposition

Acute leukemia, encompassing the majority of leukemia diagnoses, is commonly associated with chromosomal translocations that generate oncogenic fusion genes. Among these, TEL-AML1 resulting from a translocation between chromosomes 12 and 21 is well-characterized. This alteration impairs the tumor-suppressor role of TEL and modifies AML1 function in hematopoietic differentiation, facilitating malignant transformation. Inherited syndromes such as Down syndrome, Bloom syndrome, and Fanconi anemia are also linked to increased leukemia susceptibility. For instance, in Down syndrome, trisomy 21 leads to overexpression of genes like cystathionine gamma-lyase (CTSL), which may influence sensitivity to chemotherapy, indicating potential personalized therapeutic strategies [12][13].

## 2. The Role of A20 in the Incidence and Progression of Acute Leukemia

### 2.1 Overview of the A20 Gene

The A20 gene, located on chromosome 6q23 and also known as TNFAIP3 (tumor necrosis factor α-induced protein 3), spans approximately 16 kb and encodes a full-length mRNA of about 4 kb. The open reading frame (ORF) includes a 66 bp 5′-UTR and a 2001 bp 3′-UTR. A20 contains seven Cys2/Cys2-type zinc finger motifs at its C-terminal, allowing it to function as both a deubiquitinating enzyme and an E3 ubiquitin ligase. These motifs facilitate dimerization and protein-protein interactions, which are critical in regulating protein stability and immune responses. A20 acts as a key negative regulator of the NF-κB signaling pathway, thereby playing a pivotal anti-inflammatory role [14][15]. Its expression is inducible under cellular stress or immune stimuli, and its dysfunction has been implicated in various pathological states including autoimmune diseases, chronic inflammation, and tumorigenesis [16][17].

Recent research has further expanded A20’s functional profile beyond NF-κB inhibition. It is now recognized for its involvement in the regulation of cell death mechanisms (apoptosis, necroptosis, pyroptosis), autophagy, and cellular metabolism, underlining its importance in immune homeostasis and disease progression [18].

### 2.2 Association Between A20 and Acute Leukemia

Several studies have identified mutations in the A20 gene among patients with T-cell acute lymphoblastic leukemia (T-ALL), particularly in adults. These mutations are associated with poor prognosis and may serve as biomarkers for risk stratification [14]. Additionally, decreased A20 expression has been reported in adult ALL samples [19]. In acute myeloid leukemia (AML), A20 expression patterns differ significantly between patients in complete remission and those with relapsed or drug-resistant disease, suggesting a potential role in chemoresistance [20]. Collectively, these findings suggest that A20 may serve not only as a prognostic indicator but also as a potential therapeutic target in the management of acute leukemia. It is important to note that some mechanisms of A20 action are primarily elucidated in non-leukemic contexts. Their relevance to acute leukemia remains to be fully established and warrants further investigation.

## 3. Function of A20 in Acute Leukemia

**Figure-1:**
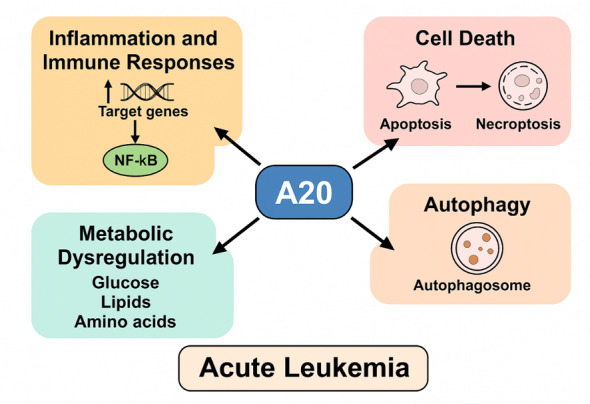
Figure 1. Schematic representation of A20 function in inflammation, immune regulation, and leukemia pathogenesis

**Table:** Table1. Functional Roles of A20 in Cell Death Pathways, Autophagy, and Metabolic Dysregulation in Leukemia

**Biological Process **	**Associated Mechanism/Pathway **	**Role of A20 **	**Evidence in Leukemia/Related Conditions **
**Apoptosis**	NF-κB, RIPK1, Caspase-8, TNFR1	Inhibits NF-κB and caspase-dependent apoptosis via RIPK1 and TRADD interactions	Suppresses apoptosis and promotes survival in B-ALL and AML
**Necroptosis**	RIPK3, Necrostatin-1	Suppresses RIPK3-mediated necroptosis; required for necrostatin-1 anti-necroptotic effects	Protective role in microglia, fish kidney cells, and Alzheimer’s disease
**Pyroptosis**	NLRP3 Inflammasome, NF-κB	Inhibits pyroptosis by downregulating NLRP3 and IL-1β via NF-κB suppression	No direct leukemia evidence, but may impact leukemogenesis via inflammation
**Autophagy**	Beclin1, ATG16L1, mTOR, TRAF6	Negatively regulates autophagy through Beclin1 deubiquitination and mTOR signaling modulation	Affects CD4+ T cell proliferation in ATL; maintains epithelial barrier
**Metabolic Dysregulation **	Glut1, Glycolysis, Mitochondrial Respiration	Modulates glucose and lipid metabolism; promotes glucose uptake and mitochondrial activity	Enhances glucose metabolism in T-ALL; alters lipid metabolism in hepatocytes

### 3.1 A20 Regulates Inflammation and Immune Responses

Inflammation is a biological response triggered by harmful stimuli such as infections or tissue injury. This process involves multiple signaling cascades, with the NF-κB pathway playing a central role in converting extracellular signals into cellular responses. External stimuli like IL-1β, TNF-α, and lipopolysaccharide (LPS) activate upstream molecules such as transforming growth factor β-activated kinase 1 (TAK1) and the IκB kinase (IKK) complex, leading to phosphorylation and degradation of IκBα. Consequently, NF-κB translocates to the nucleus, promoting the transcription of target genes.

A20 (also known as TNFAIP3) is typically expressed at low or undetectable levels under physiological conditions. However, upon NF-κB activation, its expression is induced via two NF-κB binding sites on its promoter. A20 is a key negative regulator of the NF-κB pathway, and its dysfunction has been implicated in various inflammatory and autoimmune disorders, including inflammatory bowel disease, rheumatoid arthritis, chronic obstructive pulmonary disease, systemic lupus erythematosus, and atherosclerosis [21][22].

In cancer, A20 expression exhibits a complex role. For instance, in colorectal cancer, elevated A20 levels are associated with reduced infiltration of immune effector cells such as CD8+ T cells and macrophages, correlating with a poor prognosis [23]. It is essential for the development and function of dendritic cells, B cells, T cells, and macrophages [24].

In acute myeloid leukemia (AML), Zhang X. et al. demonstrated that A20 inhibition enhances the expression of costimulatory molecules and pro-inflammatory cytokines in leukemia-derived dendritic cells, suggesting that A20 suppresses the immunogenicity and maturation of these cells [25]. Furthermore, the migration of acute lymphoblastic leukemia (ALL) cells in response to interleukin-6 is also A20-dependent [19]. These findings suggest that A20 contributes to leukemogenesis and disease progression through its modulation of inflammatory signaling. These findings suggest that A20 contributes to leukemogenesis and disease progression through its modulation of inflammatory signaling. The role of A20 in modulating inflammation, immune responses, cell death, autophagy, and metabolic regulation in the context of acute leukemia is illustrated in Figure-1.

### 3.2 A20 Regulates Cell Death

Programmed cell death (PCD) includes several tightly regulated processes such as apoptosis, necroptosis, and pyroptosis. Apoptosis involves caspase-dependent pathways mediated by either death receptors or mitochondrial signals. A20 functions as a dual inhibitor of NF-κB signaling and apoptosis. It interacts with receptor-interacting protein kinase 1 (RIPK1) via its ZnF7 domain, affecting caspase-8 expression and thereby inhibiting apoptosis in intestinal epithelial cells [26]. A20 also modulates tumor necrosis factor receptor 1 (TNFR1), TNFR1-associated death domain protein (TRADD), and RIPK1, thereby suppressing TNF-induced apoptosis, as observed in Crohn’s disease.

Elevated A20 expression has been documented in patients with B-cell ALL compared to healthy individuals [27]. In vitro studies confirm A20 upregulation in several ALL-cell lines, and A20 knockdown results in decreased proliferation, increased apoptosis, and enhanced chemosensitivity [28]. Additionally, Toxoplasma gondii infection, common in T-cell ALL, induces A20 expression and suppresses NF-κB signaling, promoting apoptosis in leukemic T cells [29]. In AML, ectopic expression of A20 supports the survival and differentiation of THP-1 cells, while inhibiting apoptosis [30]. These findings suggest a critical role for A20 in leukemic cell survival and chemoresistance.

A20 also influences necroptosis, a caspase-independent, calpain-mediated form of inflammatory cell death. For example, melatonin inhibits RIPK3-mediated microglial necroptosis by upregulating A20, and A20 is essential for the anti-necroptotic effects of necrostatin-1 [31]. Lysosomal degradation of A20 has been linked to endothelial necroptosis in Alzheimer’s disease. Resveratrol also alleviates chlorothalonil-induced necroptosis in fish kidney cells through A20 upregulation [32][33]. Thus, A20 appears to suppress both apoptotic and necroptotic pathways and holds potential as a therapeutic target.

Pyroptosis, another form of inflammatory cell death, is mediated by the NLRP3 inflammasome and involves NF-κB signaling. A20 inhibits pyroptosis by downregulating NLRP3 and pro-IL-1β expression via suppression of NF-κB. However, A20-deficient mice develop spontaneous arthritis independent of NF-κB, indicating additional mechanisms in A20’s regulation of pyroptosis [34][35]. Although direct links between A20 and pyroptosis in acute leukemia have not been reported, given the close interplay between pyroptosis and inflammation, A20 may influence leukemogenesis through pyroptotic mechanisms.

### 3.3 A20 Regulates Autophagy

Autophagy is a conserved, cathepsin-dependent process that recycles cellular components via lysosomal degradation. Two primary pathways mediate autophagy: the Atg5-Atg12 pathway and the Beclin1 pathway. A20 negatively regulates autophagy by deubiquitinating lysine 117 of Beclin1 through its OTU domain. It also interacts with ATG16L1 to maintain epithelial barrier function. Under hypoxia, A20 suppresses autophagy by modulating TRAF6 ubiquitination and thereby inhibits osteoclastogenesis.

In ankylosing spondylitis, A20 enhances early autophagy by stabilizing mTOR-interacting proteins [36]. Matsuzawa et al. reported that A20 limits mTOR signaling through ZnF7-mediated ubiquitination, promoting autophagy. CD4+ T cells lacking A20 exhibit mitochondrial accumulation and oxidative stress, leading to impaired proliferation, whereas A20 overexpression enhances CD4+ T cell survival and proliferation [37][38].

Clinical observations show elevated numbers of mature CD4+ T cells in patients with adult T-cell leukemia [39]. Given the regulatory role of A20 in T cells and autophagy, further studies should explore whether A20 modulates leukemogenesis through autophagy.

### 3.4 A20 is Associated with Cellular Metabolic Dysregulation

Acute leukemia is often accompanied by profound metabolic disturbances, including aberrant glucose, lipid, and amino acid metabolism. These dysregulations contribute to leukemic cell proliferation, treatment resistance, and relapse [40][41][42][43].

A20 influences metabolic pathways at multiple levels. Damrauer et al. demonstrated that A20 overexpression alters fatty acid metabolism in murine hepatocytes [44]. A20 also modulates glucose metabolism; mice lacking A20 are protected against diet-induced obesity and insulin resistance [45]. In hepatic malignancies, A20 regulates glycolysis and mitochondrial respiration [46]. Furthermore, in acute T-cell leukemia, A20 promotes glucose uptake and oxygen consumption by regulating Glut1, a key glucose transporter. These findings suggest that A20 contributes to metabolic reprogramming in leukemia, although direct evidence in human AL remains limited. Functional roles of A20 in cell death pathways, autophagy, and metabolic dysregulation in leukemia are summarized in Table-1 [47].

## Conclusion

The monitoring of prognostic biomarkers and the screening of new therapeutic targets are very important for the early diagnosis and effective treatment of acute leukemia. With the continuous in-depth research on the biology of acute leukemia, more and more biomarkers are incorporated into classification schemes and clinical treatments. In this paper, we summarized the mechanism and research progress of A20 in AL, and found that A20 can not only regulate inflammatory response, but also play an important role in regulating cell death and cell metabolism. More importantly, A20 plays an important role in leukemia immune escape. Exploring the function and pathogenic mechanism of A20 in acute leukemia will provide a new theoretical basis and therapeutic target for the treatment of acute leukemia.

## Conflict of Interest

The authors declare that they have no conflict of interest.
